# Is there a difference between the incidence of subtypes of tibial plateau fractures between six different level 1, level 2 and level 3 trauma centers in the Netherlands?

**DOI:** 10.1186/s12891-025-08383-8

**Published:** 2025-02-19

**Authors:** N. van der Gaast, R. L. Jaarsma, M. J. R. Edwards, J. N. Doornberg, E. Hermans, the Trauma Platform Collaboration, Jellina Huitema, Nick Assink, Frank IJpma, Claartje Meijs, Jan Duijff, Edo Hekma, Patrick Moerbeek, Marcel de Bruin, Ivo Beetz, Bart van Wijk, Kirsten Peperkamp, Diederik Smeeing

**Affiliations:** 1 Department of Trauma Surgery, Radboud University Medical Center, Geert Groteplein Zuid, Nijmegen, 6525 GA the Netherlands; 2 Department of Orthopaedic & Trauma Surgery, Flinders Medical Centre, Flinders University, Adelaide, Australia; 3 Department of Orthopaedic Surgery, University Medical Center Groningen, Groningen, the Netherlands

**Keywords:** Tibia plateau fractures, Proximal tibia fracture, Tibial plateau

## Abstract

**Purposes:**

Tibial plateau fractures can present as different subtypes due to variation in patient characteristics and trauma mechanisms. Insight in the fracture pattern and classification is critical for adequate treatment.This study aims to assess the incidence of tibial plateau fracture subtypes among various levels of trauma centers in the Netherlands, to explore potential variations in fracture patterns and fracture classifications between these centers.

**Methods:**

Over a one-year period data was collected from six Dutch trauma centers representing different levels of trauma care. Fractures were classified using both Schatzker and Luo’s systems. Interobserver agreement was calculated to evaluate the consistency of fracture classification among surgeons.

**Results:**

We included 115 patients treated for a tibial plateau fracture across six different hospitals. The mean patient age was 54 years, ranging from 17 to 85 years. Differences in fracture incidence and mechanisms of injury across trauma centers were revealed; Level 1 trauma centers treated a higher proportion of high-energy trauma cases, predominantly Schatzker 6 fractures, while level 2 and 3 centers dealt with more low-energy traumas, particularly Schatzker 2 fractures. Interobserver agreement for both classification systems was moderate, indicating challenges in classifying tibial plateau fractures accurately.

**Conclusion:**

This study sheds light on the diverse distribution of subtypes of tibial plateau fractures in Dutch trauma centers. Level 1 centers are treating younger, high-energy trauma patients, whereas level 2 and 3 centers handle more low-energy traumas, predominantly Schatzker 2 fractures.

**Trial registration:**

METC Oost-Nederland: 2021–13,184.

**Supplementary Information:**

The online version contains supplementary material available at 10.1186/s12891-025-08383-8.

## Introduction

In the Netherlands, the trauma system is stratified into three levels, each offering varying degrees of specialized care and resources. When comparing the Dutch trauma system to the United States, the differences mirror variances in health care infrastructure, the allocation of resources and notably, different geographical considerations. The Dutch system consists of three levels of trauma centers, while the United States uses a five-level trauma center classification system. However, the regionalization of the Dutch trauma system tends to follow the lead of the United States’ model of a statewide trauma system [1, 2]. In the Dutch trauma system, the government accredited a total of 10 level 1 trauma centers in 1999. They focus on treatment of polytraumatized patients and are characterized by advanced capabilities, including multiple specialized trauma rooms, the availability of specialties such as cardiothoracic and neurosurgery and advanced facilities such as cell-saving possibilities and fully equipped intensive care units (ICU), which is comparable to the level 1 criteria of the United States. Level 2 trauma centers are less equipped than level 1 trauma centers and focus of treatment is given to complex monotrauma injuries, e.g. complex tibial plateau fractures. This is comparable to level 2 and 3 trauma centers in the United States. Lastly, level 3 centers have limited capabilities, e.g. lack of ICU capabilities and major surgical facilities, which is in line with level 4 and level 5 trauma centers in the United States [1, 3–5].

Tibial plateau fractures account for approximately 1–2% of all fractures in adults and they can be challenging to treat and show a high complication rate [6–12].Typically, these fractures exhibit a bimodal distribution; Tibial plateau fractures are more commonly associated with high-energy trauma in the younger population, leading to complex fracture patterns due to high-energy forces. In elderly, but still active patients, osteoporosis becomes a contributing factor, potentially leading to complex fracture patterns following low-energy accidents [7, 13–15]. Complex tibial plateau fractures require a detailed assessment of the fracture with a thorough analysis of patient characteristics to select the most fitting treatment approach. However, this selection of the most appropriate treatment remains a challenge for surgeons and continues to be a topic of debate in current literature [7, 13, 15–17].

Various classification systems have emerged to address tibial plateau fractures and its fracture treatment by distinct injury mechanisms and fracture patterns observed through radiographs and CT scans. Schatzker et al. [18] introduced a classification system based on radiographic assessments, dividing the tibial plateau into lateral and medial condyles while distinguishing between unicondylar and bicondylar fractures. This system has become the prevailing standard in fracture classification. However, the advent of Computed Tomography (CT) scans unveiled a different dimension of tibial plateau fractures. Based on axial CT images, Luo’s three-column concept [19] proposed a segmentation for the tibial plateau into a lateral, medial and posterior column. In a systematic review, Millar et al. [20] assessed the intra- and interobserver reliability of five classification systems. The more frequently used Schatzker classification demonstrated moderate intra- and interobserver agreement, whereas Luo’s three-column concept, being a simpler classification system, exhibited higher reliability.

The incidence of tibial plateau fractures presumably varies across different trauma centers nationally and worldwide; for level 1 trauma centers one would expect to encounter more patients with severe injuries resulting from high-energy trauma (HET), whereas for level 2 and level 3 trauma centers more single limb and potentially low-energy trauma (LET) could be expected. However, do these expectations translate to the type of tibial plateau fracture that is seen in each of the three levels of trauma centers in the Netherlands? To the best of the authors’ knowledge, there are no studies comparing the incidence of sub-classifications and mechanism of injury of tibial plateau fractures between level 1, level 2, and level 3 trauma centers.

The objectives of this study are:

1. To assess the differences in the mechanism of injury (LET vs. HET) that caused a tibial plateau fracture between two level 1, two level 2 and two level 3 trauma centers.
2. To evaluate the differences in incidence of subtypes of tibial plateau fractures in six distinct level 1, level 2, and level 3 trauma centers.
3. To assess the interobserver agreement for the classification of tibial plateau fractures among surgeons.

We hypothesize that there will be differences in the incidence of different subtypes of tibial plateau fractures caused by different trauma mechanisms across level 1, level 2 and level 3 trauma centers and a moderate interobserver agreement for the assessment of tibial plateau fractures by different surgeons.

## Methods

This study was approved by the institutional reviewing board of the main study center (METC Oost-Nederland: 2021–13184). Following approval, we invited six hospitals, representing all three levels of trauma care according to the Dutch Trauma Stratification system, to take part in this study (Radboudumc, Nijmegen - Level 1; UMCG, Groningen – level 1; Rijnstate Hospital, Arnhem – Level 2; Slingeland Hospital, Doetinchem – Level 2; Rivierenland, Tiel – Level 3; Maasziekenhuis Pantein, Boxmeer – Level 3). Each participating hospital underwent a review of the study protocol by their institutional reviewing board, data-sharing agreements were finalized and informed consent was retrieved. The procedures used in this study adhere to the tenets of the Declaration of Helsinki.

This study included adult patients who suffered from tibial plateau fractures across these hospitals from January 1st, 2019, until December 31st 2019. Exclusion criteria encompassed no informed consent for participation in this study, tibial eminence avulsion fractures, congenital bone disorders, pathological fractures and the absence of adequate radiographs or CT-scans, as this was deemed necessary for the gold standard to correctly classify the included fractures. In each participating hospital, radiographs and CT-scans were collected and anonymized. All fractures were classified according to the Schatzker classification and Luo’s three-column concept by a surgeon from each center. The gold standard for fracture classification was established by a panel of three experienced surgeons from a level 1 trauma center. A consensus meeting was conducted to ensure agreement on all included fractures. The interobserver agreement was calculated for the fractures included from five hospitals comparing the classification of the trauma surgeon from each participating center to the gold standard. The fractures from the level 1 trauma center that initiated this study were excluded as they were only classified by the panel of three experienced surgeons. Lastly, the accuracy was calculated, comparing the classifications of participating surgeons to our gold standard.

Patient demographics, such as age, sex and mechanism of injury were collected. Mechanism of injury was divided into seven different categories: Bicycle accident, Motor Vehicle Accident, Crush Injury, Fall from Height, Fall from Standing, Sports Injury and other (i.e. fall from kitchen step). These groups were divided into LET (Bicycle accident, Fall from Standing and Sports Injury and Other) and HET (Motor Vehicle Accident, Crush Injury and Fall from Height).

Data sharing occurred through a Digital Research Environment, with personal logins for each center. All radiographs and were uploaded in a secured storage. Patient characteristics and fracture classifications were documented through Castor EDC ©, with a personal login for each participating center.

### Statistical analysis

IBM SPSS (version 28.0.1, Chicago, United States) was used for the statistical analysis of this study. Patient characteristics were presented as frequencies and percentages for categorical variables and as means and ranges for continuous variables. Visual inspection of histograms was performed to evaluate the distribution of patient characterics. The continuous and categorical variables of all groups were compared to each other using an independent samples t-test and a chi-square test respectively. Classifications and the mechanism of injury were presented as frequencies and percentages per level (1,2 or 3) trauma center for each classification system used. The interobserver agreement was calculated using Cohen’s kappa and to interpret the interobserver agreement, the proposed division of Landis and Koch [21] was used. A kappa between 0.01 and 0.20 reflects slight agreement, between 0.21 and 0.40 reflects fair agreement, between 0.41 and 0.60 reflects moderate agreement, between 0.61 and 0.80 reflects substantial agreement, and greater than 0.81 reflects almost perfect agreement.

## Results

### Patient demographics

A total of 115 patients with tibial plateau fractures, treated between January 1st and December 31st, 2019, across six different hospitals, were included in this study after providing informed consent. Among them, 36 patients were treated in a level 1 trauma center (Radboudumc, Nijmegen and UMCG, Groningen), 64 patients were treated in a level 2 trauma center (Rijnstate Hospital, Arnhem and Slingeland Hospital, Doetinchem) and 15 patients in a level 3 trauma center (Rivierenland Hospital, Tiel and Maasziekenhuis Pantein, Boxmeer). The data were confirmed to follow a normal distribution. The mean age was 54 years (range: 17–85 years) (Table 1). There was a significant difference between the age of patients from level 1 trauma centers and level 2 trauma centers (48 years vs. 57 years, *p* = 0.009). No significant differences were found when comparing the age of level 1 and 3 trauma centers and level 2 and 3 trauma centers respectively (*p* = 0.12 and *p* = 0.76). In terms of gender distribution, 43.5% were male and 56.5% were female. No significant differences were observed in gender distribution between groups. There were no missing data.

**Table 1:** Age and mechanism of injury for different levels of trauma centers

	Level 1		Level 2		Level 3		Total	
Mean Age (Range)	48 (17–85)*		57 (20–85)*		56 (32–82)		54 (17–85)	
Sex								
Male	50.0% (18)		40.6% (26)		46.7% (7)		43.5% (50)	
Female	50.0% (18)		59.4% (38)		53.3% (8)		56.5% (65)	
Mechanism of Injury	N	%	N	%	N	%	N	%
Bicycle Incident	11	30.6	14	21.9	2	13.3	27	23.5
Motor Vehicle Accident	10	27.8	16	25.0	2	13.3	28	24.3
Crush Injury	3	8.3	0	0.0	1	6.7	4	3.5
Fall From Height	3	8.3	4	6.3	2	13.3	9	7.8
Fall From Standing	2	5.6	19	29.7	4	26.7	25	21.7
Sports Injury	2	5.6	5	7.8	3	20.0	10	8.7
Other	5	13.9	6	9.4	1	6.7	12	10.0
Total	36	97.2	64	100	15	100	115	100.0

*Significant difference (*p* = 0.009)

### Mechanism of injury

The mechanism of injury of each patient is outlined in detail in Table 1 and classified into low energy trauma (LET) and high-energy trauma (HET), as illustrated in Table 2. In level 1 trauma centers, the proportion of HET exceeded that of level 2 and 3 trauma centers, whereas level 2 and 3 trauma centers demonstrated a higher percentage of LET cases. Level 2 and level 3 trauma centers exhibited comparable numbers of LET and HET. There were no significant differences between each group (Tables 1 and 2).

**Table 2:** LET vs. HET for different levels of trauma centers

	Level 1		Level 2		Level 3		Total	
	N	%	N	%	N	%	N	%
Low Energy Trauma (LET)	20	55.6	44	68.7	10	66.7	74	64.3
High Energy Trauma (HET)	16	44.4	20	31.3	5	33.3	41	35.7
Total	36	100.0	64	100.0	15	100.0	115	100.0

### Incidence of subtypes Schatzker classification

The incidences of subtypes of the Schatzker classification, according to our participating hospitals and our expert panel can be found in Table 3. Overall, the most common subtype of the Schatzker classification is type 2, followed by type 4 and 6. According to the participating centers, more tibial plateau fractures were classified as a Schatzker type 1 (Table 3).

**Table 3:** Incidence of subtypes of tibial plateau fractures in level 1, 2 and 3 trauma centers – schatzker classification

	Level 1		Level 2		Level 3		Total	
	Participating center N (%)	Gold Standard N (%)	Participating centers N (%)	Gold Standard N (%)	Participating centers N (%)	Gold Standard N (%)	Participating centers N (%)	Gold Standard N (%)
Schatzker 1	2 (10.0)	3 (8.3)	14 (21.9)	5 (7.8)	1 (6.7)	1 (6.7)	17 (17.2)	9 (7.8)
Schatzker 2	6 (30.0)	9 (25.0)	22 (34.4)	29 (45.3)	5 (33.3)	8 (53.3)	33 (33.3)	46 (40.0)
Schatzker 3	2 (10.0)	4 (11.1)	4 (6.3)	3 (4.7)	2 (13.3)	0 (0.0)	8 (8.1)	7 (6.1)
Schatzker 4	4 (20.0)	9 (25.0)	9 (14.1)	6 (9.4)	3 (20.0)	2 (13.3)	16 (16.2)	17 (14.8)
Schatzker 5	1 (5.0)	1 (2.8)	5 (7.8)	7 (10.9)	2 (13.3)	3 (20.0)	8 (8.1)	11 (9.6)
Schatzker 6	5 (25.0)	10 (27.8)	10 (15.6)	14 (21.9)	2 (13.3)	1 (6.7)	17 (17.2)	25 (21.7)
Total	20	36	64	64	15	15	99	115

### Incidence of subtypes Luo’s three-column concept

The incidences of subtypes of Luo’s three-column concept, according to our participating hospitals and our expert panel can be found in Table 4. The most common fracture type was a lateral and posterior fracture of the tibial plateau across all levels of trauma centers, followed by a three-column fracture, involving the lateral, medial and posterior column (Table 4).

**Table 4:** Incidence of subtypes of tibial plateau fractures in level 1. 2 and 3 trauma centers – Luo’s three-column concept

	Level 1		Level 2		Level 3		Total	
	Participating centers N (%)	Gold standard N (%)	Participating centers N (%)	Gold standard N (%)	Participating centers N (%)	Gold standard N (%)	Participating centers N (%)	Gold standard N (%)
Lateral	5 (13.9)	2 (5.6)	7 (10.9)	15 (23.4)	4 (26.7)	0 (0.0)	16 (16.2)	17 (14.8)
Medial	1 (2.8)	4 (11.1)	0.0	1 (1.6)	2 (13.3)	1 (6.7)	3 (3.0)	6 (5.2)
Posterior	1 (2.8)	2 (5.6)	7 (10.9)	3 (4.7)	1 (6.7)	0 (0.0)	9 (9.1)	5 (4.3)
Lateral + Medial	0 (0.0)	0 (0.0)	0 (0.0)	0 (0.0)	0 (0.0)	1 (6.7)	0 (0.0)	1 (0.9)
Lateral + Posterior	6 (16.7)	15 (41.7)	27 (42.2)	20 (31.3)	6 (40.0)	10 (66.7)	39 (39.4)	45 (39.1)
Medial + Posterior	1 (2.8)	3 (8.3)	9 (14.1)	5 (7.8)	0 (0.0)	1 (6.7)	10 (10.1)	9 (7.8)
Lateral + Medial + Posterior	6 (16.7)	10 (27.8)	14 (21.9)	20 (31.3)	2 (13.3)	2 (13.3)	22 (22.2)	32 (27.8)
Total	20	36	64	64	15	15	99	115

### Differences in classification

The interobserver agreement for the Schatzker classification and Luo’s three-column concept and each level trauma center can be found in Table 5. The overall interobserver agreement for the Schatzker classification is 0.52. The overall interobserver agreement for Luo’s three-column concept is 0.41. According to the interpretation of Landis and Koch [21], both classification systems reflect a moderate agreement. The overall accuracy for the Schatzker classification was 62.6% and 60.6% for Luo’s three-column concept. (Table 5) When classifying fractures according to the Schatzker classification, the primary disparity in classification occurred between the classification of Schatzker 1, Schatzker 2 and Schatzker 3 fractures. Additionally, Schatzker 2 fractures were occasionally misclassified as Schatzker 5 fractures and vice versa. Concerning Luo’s three-column concept, the main differences in classification were lateral column fractures to lateral and posterior column fractures (Supplementary file 1).

**Table 5:** Interobserver agreement of all fractures for Schatzker classification and Luo’s three-column concept

	Number of fractures	Interobserver agreement (Cohen’s Kappa)	Agreement according to Landis and Koch	Accuracy
Schatzker classification	99	0.52	Moderate	62.6%
Level 1 Level 2 Level 3	20 64 15	0.75 0.45 0.47	Substantial Moderate Moderate	80.0% 59.4% 60.0%
Luo’s Three-column concept	99	0.41	Moderate	60.6%
Level 1 Level 2 Level 3	20 64 15	0.54 0.49 0.15	Moderate Moderate Slight	65.0% 62.5% 40%

## Discussion

This study aimed to evaluate the differences in trauma mechanism and incidence of subtypes of tibial plateau fractures across different trauma centers in the Netherlands. The incidence of subtypes of tibial plateau fractures was notably different between the participating trauma centers. Level 1 trauma centers exhibited a higher prevalence of Schatzker 4 and Schatzker 6 tibial plateau fractures compared to level 2 and level 3 trauma centers. In level 2 and level 3 trauma centers, the highest incidence of Schatzker 2 fractures was found. The variations in patient populations and trauma mechanisms across Level 1, Level 2, and Level 3 trauma centers, translating into different incidences of subtypes of tibial plateau fracture types, are confirmed by our analysis of patient age and injury mechanisms. Level 1 trauma centers exhibited higher proportions of high-energy trauma compared to level 2 and 3 trauma centers, as could be expected. The interobserver agreement was overall moderate for both the Schatzker classification and Luo’s three-column concept.

### Interpretation of results

The incidence of different subtypes of tibial plateau fractures exhibit similarities, yet display differences in their occurrence among different populations and mechanisms of injury when comparing to recent literature. Albuquerque et al. [22] conducted a retrospective analysis involving 239 surgically treated tibial plateau fracture at a level 1 trauma center in Brazil, revealing a mean age of 44.5 years, with 70% of patients being male. Fall from height and motorcycle accidents were the most common mechanisms of injury, consistent with our findings from patients treated at level 1 trauma centers in the Netherlands, where a mean age of 48 years and and motor vehicle accidents and bicycle accidents being the predominant mechanisms of injury. Elsoe et al. [9] and Wennergren et al. [10] both reported the incidence of tibial plateau fractures treated in different hospitals in Denmark and Sweden, respectively. Both studies utilized the AO/OTA classification, identifying AO/OTA 41B3 and AO/OTA 41C3 fractures as the most common fractures, which could be compared with Schatzker 2 and Schatzker 6 fractures respectively. They observed a tendency towards younger males with more severe trauma mechanisms and older females with lower energy traumas, echoing our results, indicating a higher incidence of high-energy trauma in level 1 trauma centers, where patients are significantly younger on average. As of the Dutch trauma system, it is suggested that young patients with high-energy injuries should be directed to level 1 trauma centers, while elderly patients with low-energy traumas are better treated at level 2 and 3 centers. The complex monotraumas (for example open fractures or with additional vascular injuries) are referred to a level 1 trauma center when deemed necessary. This observation aligns with the typical referral patterns in the Netherlands, indicating that current hospital referrals for tibial plateau fractures are effective.

Donovan et al. [23] performed a prospective analysis over a 10-years period at a major trauma center in the UK, focussing on patients > 60 years with tibial plateau fractures. Most patients were female (73%), the mean age was 74 years and a fall from standing was the primary mechanism of injury in this study (71%). Schatzker type 2 fractures were again the most common (32%), followed by Schatzker 6 (18%) and Schatzker 1 (17%). In our study, the overall incidence of Schatzker 2 fractures, according to our gold standard, was higher (40.0%) and the incidence of Schatzker 1 fractures was lower (7.8%) When comparing the classification of our participating surgeons to our gold standard, the most common disparity in classification compared to the gold standard was a difference in classification between Schatzker 1 and 2. Due to overlapping characteristics, the radiographic presentation of Schatzker 1 and 2 fractures may appear similar, making it challenging to distinguish. When using CT imaging as the gold standard for classification, as in our study, more fractures may be classified as Schatzker 2 fractures due to the more elaborate view provided by CT. This emphasizes the difficulties in classifying tibial plateau fractures and could be the cause of the differences in incidence of Schatzker 1 and Schatzker 2 fractures. Their study also reported on the classification according to Luo’s Three-column Concept, with ‘Lateral and Posterior’ fractures and ‘Three-Column’ fractures being predominant, aligning with our results.

Lastly, literature demonstrates moderate to substantial interobserver agreement among surgeons classifying fractures according to the Schatzker classification when using both radiographs and CT [20, 24], consistent with our results. Regarding Luo’s three-column concept, the interobserver agreement is varying between moderate to substantial in existing literature [20]. Our study corresponds the lower end of this spectrum, indicating only moderate agreement. This highlights the variability of interpreting the Schatzker classification and Luo’s three-column concept, regardless the improvements of imaging methods, using Three-Dimensional (3D) CT and 3D printed models [20, 24, 25]. The Schatzker classification, originally intended for the use of plain radiographs, faces limitations in the era of 3D imaging facilitated by CT scans. While this might improve more thorough insights into the fractures, this could be the cause of misclassification and different treatment plans due to this more comprehensive view. The misclassification of fractures poses a significant problem, which could potentially lead to different treatment of similar subtypes of tibial plateau fractures. Numerous studies explore the classification and treatment of tibial plateau fractures. However, there is a lack of research examining how variations in classification impact treatment strategies and patient outcomes [7]. Luo’s Three-column Concept is based on axial CT scans. Despite the focus on more advanced imaging using CT, the interobserver agreement for this classification system is comparable to the Schatzker classification [20, 26]. One should search for more homogeneity in fracture classification to create more consistency in tibial plateau fracture treatment. Integrating deep learning algorithms could significantly improve fracture detection and classification while enhancing the consistency of treatment plans by predicting outcomes and guiding the treatment process accordingly [27–30]. However, integrating these algorithms safely into clinical practice remains a considerable challenge.

### Study limitations

This study has a couple of limitations. Firstly, the data from six trauma centers within a specific time frame of a year might limit the generalizability of the findings to other populations and healthcare settings. Ideally, one could use a national joint registry, however, these large registers often lack specific fracture classifications. Therefore, we aimed to focus on our regional presentation of tibial plateau fractures. Second, the study sample of 115 patients with tibial plateau fractures might be relatively small for assessing rare fracture subtypes, such as Schatzker 1 and Schatzker 5 fractures. Due to lack of informed consent of patients in participating centers, the number of included patients was lower. This could affect the precision and reliability of the incidence estimates in detail. However, when comparing our numbers to current literature, no major differences were found. Lastly, this study has not accounted for the impact of the differences in classification on the choice of treatment and the patient-related outcomes. Changing classifications from unicondylar to bicondylar fractures could potentially influence the choice of treatment.

## Conclusion

This study provides an overview on the mechanisms of injury of tibial plateau fractures across different level 1, level 2 and level 3 trauma centers in the Netherlands and incidence of subtypes of tibial plateau fractures within these centers. Level 1 trauma centers are treating more high-energy trauma in a younger population, whereas level 2 and 3 trauma centers show a tendency towards low energy trauma with a higher incidence of unicondylar Schatzker 2 tibial plateau fractures. Our results demonstrate that classification of fractures is a difficult task with multiple interpretations, resulting in an overall moderate interobserver agreement, which can potentially lead to inconsistencies in surgical treatment.

## Supplementary Information

Supplementary Material 1.

## Data Availability

The dataset analyzed for this study is available upon reasonable request to the corresponding author.
