# Supplementary material for: Is there a difference between the incidence of subtypes of tibial plateau fractures between six different level 1, level 2 and level 3 trauma centers in the Netherlands?
Source: BMC Musculoskelet Disord. 2025 Feb 19;26:169. doi: 10.1186/s12891-025-08383-8 (PMC11837648; doi:10.1186/s12891-025-08383-8)
Supplement: Supplementary file 1 — Supplementary Material 1. [file 12891_2025_8383_MOESM1_ESM.docx]

Supplementary file 1 – Differences in classification

**Schatzker classification**

Schatzker 1 🡨🡪 Schatzker 2 : 11x

Schatzker 1 🡨🡪 Schatzker 3: 1x

Schatzker 1 🡨🡪 Schatzker 3 : 7x

Schatzker 1 🡨🡪 Schatzker 5: 1x

Schatzker 1 🡨🡪 Schatzker 6: 1x

Schatzker 2 🡨🡪 Schatzker 3 : 7x

Schatzker 2 🡨🡪 Schatzker 4 : 1x

Schatzker 2 🡨🡪 Schatzker 5 : 5x

Schatzker 2 🡨🡪 Schatzker 6: 1x

Schatzker 4 🡨🡪 Schatzker 5: 2x

Schatzker 4 🡨🡪 Schatzker 6: 2x

Unicondylar 🡨🡪 Bicondylar : 12x

**Luo’s Three-column Concept**

Lateral 🡨🡪 Lateral + Posterior : 13x

Lateral 🡨🡪 Medial : 3x

Lateral 🡨🡪 Posterior : 1x

Lateral + Posterior 🡨🡪 Three-columns : 8x

Lateral + Posterior 🡨🡪 Medial + Posterior : 1x

Lateral + Posterior 🡨🡪 Medial : 1x

Lateral + Posterior 🡨🡪 Posterior : 3x

Lateral + Medial 🡨🡪 Lateral + Posterior : 1x

Medial + Posterior 🡨🡪 Posterior : 1x

Medial + Posterior 🡨🡪 Three-columns : 5x

Posterior 🡨🡪 Three-columns: 2x
